# Nicotinic Modulation of Fast-Spiking Neurons in Rat Somatosensory Cortex across Development

**DOI:** 10.1523/ENEURO.0239-25.2025

**Published:** 2025-11-27

**Authors:** Catherine W. Haga, Jeffrey Koenig, Nathan Cramer, Ramesh Chandra, Asaf Keller

**Affiliations:** Department of Neurobiology and UM-MIND, University of Maryland School of Medicine, Baltimore, Maryland 21201

## Abstract

Signaling at nicotinic acetylcholine receptors (nAChRs) is vital for normal development of cerebral cortical circuits. These developing circuits are also shaped by fast-spiking (FS) inhibitory cortical neurons. While nicotinic dysfunction in FS neurons is implicated in a number of psychiatric and neurodevelopmental disorders, FS neurons are thought to not have nicotinic responses in adults. Here, we establish a timeline of FS neuron response to nicotine pre- and postsynaptically in primary somatosensory cortex in male and female rats. We found that nicotine increases the frequency of spontaneous synaptic inputs to FS neurons during the second postnatal week, and this effect persisted through development. In contrast, FS neurons in S1 had no postsynaptic responses to nicotine from as early as they can be reliably identified. This was not attributable to receptor desensitization, and we further revealed that FS neurons express abundant mRNA for several nAChR subunits, beginning early in development. To determine why FS neurons do not respond to nicotine despite expressing these receptors, we probed for the expression of lynx1, a negative nicotinic modulator. Lynx1 mRNA was expressed in FS neurons from early development, with expression increasing dramatically during the second postnatal week.

## Significance Statement

Signaling at nicotinic receptors is critical for development of cortical circuits. These circuits are also shaped by fast-spiking (FS) inhibitory neurons. We reveal how these developmental processes interact, by establishing a timeline of nicotinic effects on FS neurons in rats. We find that nicotine presynaptically regulates inputs to FS neurons from early development. However, FS neurons at all ages lack postsynaptic responses to nicotine, despite expressing nicotinic receptor mRNA. This might be due to the expression of lynx1, a negative nicotinic receptor modulator, which we identify in FS neurons as early as the first postnatal week. This work reveals novel aspects of development, relevant to both normal cortical function and the neuropsychiatric pathologies associated with abnormal FS neurons.

## Introduction

Acetylcholine is the endogenous neurotransmitter that acts on muscarinic and nicotinic acetylcholine receptors (nAChRs), which are expressed widely in neocortex and are important for cortical development (Fiedler et al., 1987; Dwyer et al., 2008). nAChRs are upregulated during early development and their expression in the cortex peaks by the end of the second postnatal week (Fiedler et al., 1987; Naeff et al., 1992; Tribollet et al., 2004). Development is also driven by fast-spiking, parvalbumin-expressing GABAergic neurons (FS or PV neurons), a large class of inhibitory cortical neurons with characteristic kinetic properties (Kawaguchi and Kondo, 2002; Hensch, 2005; Rudy et al., 2011). During the first postnatal weeks, the biophysical and chemical properties of FS neurons mature (Itami et al., 2007). FS neurons drive use-dependent plasticity and circuit development during developmental critical periods (Hensch, 2005; Lee et al., 2012; Kaplan et al., 2016; Choi, 2018; Rupert and Shea, 2022).

Nicotinic signaling in FS neurons is implicated in neuropsychiatric disorders that involve network dysfunction (Lin et al., 2014; Deutsch and Burket, 2020). Mutation or microdeletion of the CHRNA7 gene coding for the α7 homomeric receptor is associated with schizophrenia, intellectual disability, autism spectrum disorder, and epilepsy (Sharp et al., 2008; Lin et al., 2014; Tregellas and Wylie, 2019). Behavioral abnormalities in attention, working memory, and learning associated with these disorders have also been functionally linked to deletion of this receptor in rodent models (Lin et al., 2014; Deutsch and Burket, 2020). FS neurons are implicated in the mechanisms of these associations, as deletion of the α7 gene causes abnormal FS neuron development (Lin et al., 2014). Thus, nicotinic signaling in FS neurons is critical for normal cortical development.

There is some debate as to whether PV neurons express functional nAChRs. While some studies report that PV neurons may not express nAChRs in rodents (Couey et al., 2007; Askew et al., 2019), coexpression of both α7 and α4β2 nAChRs in PV neurons has been demonstrated in human temporal cortex (Krenz et al., 2001), and expression of β2 subunits has been identified in PV neurons in macaque visual cortex (Disney et al., 2007). However, FS neurons do not respond postsynaptically to nicotine in rodents, at least during late postnatal development through adulthood. This has been confirmed in auditory, visual, somatosensory, and prefrontal cortical areas (Couey et al., 2007; Gulledge et al., 2007; Askew et al., 2019). In contrast, whether FS neurons respond to nicotine at earlier developmental ages has not been established. Here, we address this deficiency by studying the actions of nicotine on both presynaptic and postsynaptic responses of FS neurons across development.

We establish the developmental timeline of FS neurons’ responses to nicotine in the barrel field of primary somatosensory cortex (S1), a well-established developmental model of cortical circuits and functions (Erzurumlu and Gaspar, 2012, 2020). We also investigate the expression of lynx1, an endogenous prototoxin that serves as a brake on nicotinic signaling in other cortical areas (Morishita et al., 2010), with a focus on its specific expression in fast-spiking (FS) interneurons in S1 across postnatal development.

## Materials and Methods

### Animals

All procedures adhered to the Guide for the Care and Use of Laboratory Animals and were approved by the Institutional Animal Care and Use Committee at the University of Maryland School of Medicine. Male and female Long–Evans rats from Charles River were bred in our temperature- and humidity-controlled vivarium. Animals were fed standard chow *ad libitum* and maintained on a 12 h light/dark cycle. Rats were bred in monogamous pairs with gestational timing confirmed by the presence of sperm in vaginal lavage samples. Dams were pair-housed and then single housed immediately before delivery until study endpoint. Litters were culled to 8–12 pups per dam. Preweanling offspring were used in most experiments. In some cases, pups were weaned at postnatal day 21 (P21) and socially housed until study endpoint. Preweanling (P12) and adult (P100) male and female C57BL/6 mice were used for *lynx1* RNAscope experiments.

### Slice electrophysiology

In vitro slice electrophysiology was performed as we previously described (Alipio et al., 2021). We anesthetized preweanling and adolescent rats with ketamine (40–80 mg/kg) and xylazine (5–10 mg/kg), removed their brains, and prepared 300 μm coronal sections containing primary somatosensory cortex using a Leica VT1200s Vibratome. Slices from preweanling rats up to P14 were prepared in ice-cold normal artificial cerebrospinal fluid (ACSF) containing the following (in mM): 119 NaCl, 2.5 KCl, 1.2 NaH_2_PO_4_, 2.4 NaHCO_3_, 12.5 glucose, 2 MgSO_4_·7H_2_O, and 2 CaCl_2_·2H_2_O. Slices from rats P15 and older were prepared in ice-cold NMDG ACSF containing the following (in mM): 92 NMDG, 30 sodium bicarbonate, 20 HEPES, 25 glucose, 5 sodium ascorbate, 2 thiourea, 1.25 monosodium phosphate, 2.5 potassium chloride, 3 sodium pyruvate, 10 magnesium sulfate heptahydrate, and 0.5 calcium chloride dihydrate. Slices prepared in NMDG ACSF were allowed to recover in 35–37°C NMDG ACSF for 7 min immediately after slicing before being placed in room temperature normal ACSF. All ACSF solutions were adjusted to a pH of 7.4 using HCl, and osmolarity was adjusted to 305 ± 5 mOsm/L. Solutions were saturated with carbogen (95% O_2_ and 5% CO_2_) throughout use.

We placed slices in a submersion chamber and continually perfused (2 ml/min) with normal ACSF. We obtained whole-cell patch-clamp recordings from S1 layer 4 (L4) neurons in current-clamp mode to establish their firing properties from intracellular current injections. Using a series of 1,000 ms current pulses of increasing amplitude, we recorded and analyzed the firing pattern of each neuron during the first 500 ms of stimulation. We subsequently recorded spontaneous postsynaptic currents (sPSCs) and measurements of whole-cell resistance in voltage-clamp mode (−65 mV holding potential) through pipettes (4–6 MΩ) containing the following (in mM): 120 potassium gluconate, 10 potassium chloride, 10 HEPES, 1 magnesium chloride, 0.5 EGTA, 2.5 magnesium ATP, 0.2 GTP-Tris, and 0.1% biocytin (Thermo Fisher Scientific), adjusted to pH 7.3 and 290 mOsm/L. Biocytin was included in the internal solution to allow for reconstruction and further identification of recorded cells. In some sPSC experiments, gabazine (1 μM) was added to the ACSF to suppress the effects of inhibitory inputs on the recorded cells acting on GABA-A receptors and to isolate excitatory inputs. However, even in cases where we did not block GABA-A signaling, most inhibitory currents driven by chloride would not be detectable due to a lack of driving force for this ion. For recordings in voltage-clamp mode, cells were held at −65 mV—effectively the reversal potential of chloride with our solutions—and thus we assume that recorded sPSCs are glutamatergic in nature and refer to them in Results as sEPSCs. Nicotine bitartrate dihydrate (Sigma-Aldrich) was applied in slice electrophysiology experiments by washing in with the ACSF for 3–6 min (10 µM) or directly applying ∼50 μm from the soma of recorded neurons through a glass pipette (4–6 MΩ), using a Picospritzer (20–30 µM).

We recorded baseline sEPSCs from FS neurons for a minimum of 3 min before washing in ACSF containing nicotine (10 μM) for 6 min. We compared frequency and amplitude of sEPSCs during 3 min of baseline and 3 min of nicotine application, following the initial 3 min wash-in period. Whole-cell resistance (*R*_m_) was measured in voltage-clamp mode by delivering intracellular current pulses (300 ms steps, Δ10 mV per step) and recording the steady-state current at each step. *R*_m_ was calculated with Ohm's law using the −60 mV current step. Differences in *R*_m_ before and after nicotine application were quantified as the normalized change in resistance.

In puff recording experiments, gabazine (1 μM), CNQX (20 μM), and AP5 (50 µM) were added to the ACSF to suppress synaptic activity and study somatodendritic effects of nicotine. Cells were recorded in current-clamp mode. The puff pipette was attached to a Sutter Instruments micromanipulator for positioning and pressure ejection of nicotine solution controlled with a Picospritzer. Prior to recording, the puff pipette was lowered into a region of the brain outside of the cortex and the air pressure adjusted (∼5–10 PSI) so that the pressure wave extended at least 50 µm from the tip of the pipette while causing minimal mechanical disturbance to the cells at this range. A putative FS neuron was identified, and the puff pipette placed ∼50 µm away, but aimed directly at, the cell. A second pipette was used to obtain whole-cell recordings. We checked for responses to nicotine puff application by comparing the area under the curve (AUC) of each recording 3 s prior to each puff application (baseline) and 1 s after the onset of puff application. The AUCs for the first 3–5 consecutive applications in each cell were compared using a paired *t* test with *p* < 0.05 indicating a significant response to nicotine. For cells that showed a significant response to nicotine, we report the peak change in membrane potential. Cells that did not respond to nicotine were assigned a value of 0 mV.

Series resistance was monitored in electrophysiological recordings by measuring the current evoked by a −5 mV square pulse, and recordings were discarded if resistance changed by >20%. Recordings were made with the Axon pClamp 11 Software Suite (Molecular Devices) and analyzed with Easy Electrophysiology V2 software (Easy Electrophysiology).

### Histology

After electrophysiological recordings, slices were fixed in 10% neutral buffered formalin (NBF) at room temperature for 24–48 h and stored at 4°C in PBS until processing. Slices were placed in blocking solution containing PBS, 1% BSA, and 0.3% Triton for 2 h and then incubated for 48–72 h in incubation solution (1% BSA, 0.1% Triton) containing streptavidin-conjugated Alexa 488 (1:1,000; Jackson ImmunoResearch Laboratories) and parvalbumin polyclonal antibody (1:10,000; PA1-933; Thermo Fisher Scientific). Slices were washed and incubated in a secondary antibody solution (Alexa Fluor 594 donkey anti-rabbit IgG, 1:500; Thermo Fisher Scientific) and then mounted in aqueous media and imaged using confocal microscopy. Neurons were analyzed for parvalbumin immunoreactivity, soma and dendritic morphology, and presence of dendritic spines to confirm phenotype as FS or regular spiking (RS).

### RNAscope

In situ mRNA expression levels were determined using RNAscope Multiplex Fluorescent V2 Assays (ACDBio). Animals were deeply anesthetized by intraperitoneal injection of ketamine/xylazine and transcardially perfused with ice-cold PBS followed by 10% neutral buffered formalin (NBF). Brains were extracted, fixed overnight in 10% NBF, cryoprotected in a sucrose solution, frozen, and sectioned using a cryostat (12 µM). Ready-to-use reagents from the RNAscope Multiplex Fluorescent V2 Assay Kit were used with Rn-Chrna4, Rn-Chrna7, and Rn-Pvalb probes (ACDBio) to process sections from P12 and P19 male and female rats to detect mRNA for nAChR subunits and parvalbumin. Mm-Lynx1 and Mm-Pvalb probes (ACDBio) were used to process sections from P12 and P100 male and female mice. Sections were mounted in aqueous media and imaged using confocal microscopy. In situ mRNA expression levels were quantified using Imaris 10 software (Oxford Instruments).  

### RT-qPCR 

Male and female rats were killed at P0, P4, P7, P14, and P21. Brains were extracted and dissected in ice-cold PBS to collect 14-gauge tissue punches from 1 mm sections of S1 and primary visual cortex (V1). Two brains were pooled for each region from P0 and P4 animals due to tissue volume. RNA was isolated from cortical tissue punches with TRIzol reagent (Invitrogen) and the MicroElute Total RNA Kit (Omega; catalog #R6831) with a DNase step (Qiagen; catalog #79254). RNA concentration was measured on a Nanodrop (Thermo Scientific) and 1,000 ng of mRNA from each sample was used to synthesize complementary DNA using an iScript cDNA synthesis kit (Bio-Rad; catalog #1708891). This cDNA was diluted to a concentration of 5 ng/μl, which was used to measure relative mRNA expression of lynx1 by age via quantitative PCR with PerfeCTa SYBR Green FastMix (Quantabio; catalog #95072). Primer sets used were as follows (F, R; 5′-3′): Lynx1 ACCACTCGAACTTACTTCACC, ATCGTACACGGTCTCAAAGC; GAPDH CCCACTCTTCCACCTTCGATG, TCCACCACCCTGTTGCTGTAG. All samples were run in duplicate, and samples with a difference in CT value >0.6 were excluded.  Relative quantification of mRNA expression was performed using the 2^−ΔΔCt^ method with standard protocols, using GAPDH and P21 values in each respective cortical region to normalize expression.  

### Statistical analysis

Statistical tests were conducted using GraphPad Prism 10 software. Sample sizes were determined a priori using G*Power software suite (Faul et al., 2007). Statistical significance level was set at *p* < 0.05. Effects of nicotine on individual neurons as well as group effects were analyzed. Interevent intervals (IEI) for each sEPSC were calculated, and frequency is reported as the instantaneous frequency of events or the inverse of IEIs. Effects of nicotine on frequency and amplitude of events from each neuron were separately analyzed using Mann–Whitney *U* tests (Tables 1–4).

**Table 1.** Frequency of sEPSCs by neuron (FS neurons)

Age (PND)	Sex	Gabazine	Baseline median frequency (Hz)	Nicotine median frequency (Hz)	Statistical test	*U*	*p* value	*p* < 0.05
10	Male	No	0.09	0.51	Mann–Whitney	161	<10^−4^	Yes
10	Male	No	0.66	8.72	Mann–Whitney	6,588	<10^−4^	Yes
11	Male	No	1.05	0.74	Mann–Whitney	2,617	0.12	No
11	Male	No	1.10	1.54	Mann–Whitney	9,306	0.03	Yes
11	Male	No	3.56	4.53	Mann–Whitney	116,547	0.09	No
11	Male	No	2.35	2.62	Mann–Whitney	48,401	0.08	No
13	Male	No	5.27	16.37	Mann–Whitney	4,522	<10^−4^	Yes
13	Male	No	7.05	12.52	Mann–Whitney	86,829	<10^−4^	Yes
13	Male	No	1.74	3.14	Mann–Whitney	34,142	<10^−4^	Yes
13	Male	No	6.71	7.28	Mann–Whitney	340,301	0.14	No
13	Male	No	13.07	10.54	Mann–Whitney	781,289	1.9 × 10^−3^	Yes
12	Female	No	1.05	7.37	Mann–Whitney	18,125	<10^−4^	Yes
13	Female	No	1.05	1.20	Mann–Whitney	7,808	0.92	No
14	Female	No	9.50	12.72	Mann–Whitney	725,146	<10^−4^	Yes
13	Male	Yes	8.89	9.95	Mann–Whitney	636,546	0.01	Yes
14	Male	Yes	0.65	0.56	Mann–Whitney	3,134	0.25	No
10	Female	Yes	0.58	0.56	Mann–Whitney	1,869	0.28	No
12	Female	Yes	16.99	18.59	Mann–Whitney	2,214,561	0.01	Yes
12	Female	Yes	8.78	8.40	Mann–Whitney	521,094	0.49	No
13	Female	Yes	2.00	2.00	Mann–Whitney	28,474	0.48	No
17	Male	No	0.25	0.64	Mann–Whitney	232	0.03	Yes
17	Male	No	0.45	0.97	Mann–Whitney	1,312	0.01	Yes
20	Male	No	31.49	34.51	Mann–Whitney	2,104,406	<10^−4^	Yes
20	Male	No	22.62	25.25	Mann–Whitney	1,323,782	0.61	No
24	Male	No	14.14	16.92	Mann–Whitney	665,200	<10^−4^	Yes
24	Male	No	15.46	14.90	Mann–Whitney	552,708	0.66	No
16	Female	Yes	20.70	22.77	Mann–Whitney	725,018	0.79	No
26	Female	Yes	0.77	10.28	Mann–Whitney	13,712	<10^−4^	Yes

**Table 2.** Amplitude of sEPSCs by neuron (FS neurons)

Age (PND)	Sex	Gabazine	Baseline median amplitude (pA)	Nicotine median amplitude (pA)	Statistical test	*U*	*p* value	*p* < 0.05
10	Male	No	16.24	13.86	Mann–Whitney	492	0.80	No
10	Male	No	27.51	24.46	Mann–Whitney	22,892	0.06	No
11	Male	No	15.43	15.72	Mann–Whitney	120,436	0.40	No
11	Male	No	27.72	19.91	Mann–Whitney	41,170	<10^−4^	Yes
13	Male	No	11.76	11.91	Mann–Whitney	7,065	0.68	No
13	Male	No	16.39	16.05	Mann–Whitney	121,924	0.22	No
13	Male	No	17.01	13.29	Mann–Whitney	35,516	<10^−4^	Yes
13	Male	No	13.29	13.39	Mann–Whitney	346,543	0.40	No
13	Male	No	15.55	20.79	Mann–Whitney	669,189	<10^−4^	Yes
12	Female	No	12.00	21.18	Mann–Whitney	25,027	<10^−4^	Yes
13	Female	No	10.86	13.01	Mann–Whitney	5,915	<10^−4^	Yes
14	Female	No	18.35	18.35	Mann–Whitney	856,686	0.74	No
13	Male	Yes	18.92	18.89	Mann–Whitney	677,050	0.98	No
14	Male	Yes	15.06	17.60	Mann–Whitney	2,737	0.01	Yes
10	Female	Yes	16.07	17.57	Mann–Whitney	1,827	0.16	No
12	Female	Yes	17.74	17.31	Mann–Whitney	2,262,089	0.13	No
12	Female	Yes	17.27	19.86	Mann–Whitney	457,482	<10^−4^	Yes
13	Female	Yes	16.08	15.38	Mann–Whitney	28,103	0.30	No
17	Male	No	13.72	16.57	Mann–Whitney	341	0.41	No
17	Male	No	11.13	10.56	Mann–Whitney	1,653	0.30	No
20	Male	No	19.18	18.87	Mann–Whitney	2,139,668	0.01	Yes
20	Male	No	15.91	15.77	Mann–Whitney	1,326,185	0.63	No
24	Male	No	19.68	19.10	Mann–Whitney	559,031	0.96	No
24	Male	No	16.71	16.61	Mann–Whitney	736,431	0.72	No
16	Female	Yes	19.31	18.64	Mann–Whitney	713,066	0.32	No

**Table 3.** Frequency of sEPSCs by neuron (RS neurons)

Age (PND)	Sex	Gabazine	Baseline median frequency (Hz)	Nicotine median frequency (Hz)	Statistical test	*U*	*p* value	*p* < 0.05
8	Male	No	14.14	16.92	Mann–Whitney	665,200	<10^−4^	Yes
8	Male	No	0.23	1.73	Mann–Whitney	903	<10^−4^	Yes
11	Male	No	0.93	0.96	Mann–Whitney	3,000	0.74	No
11	Male	No	6.01	7.13	Mann–Whitney	281,531	3.3 × 10^−3^	Yes
12	Male	No	2.67	4.33	Mann–Whitney	12,556	<10^−4^	Yes
12	Male	No	0.81	1.98	Mann–Whitney	2,077	<10^−4^	Yes
12	Male	No	0.24	3.04	Mann–Whitney	438	<10^−4^	Yes
12	Male	No	11.52	14.93	Mann–Whitney	1,179,051	<10^−4^	Yes
12	Male	No	3.89	8.74	Mann–Whitney	102,008	<10^−4^	Yes
13	Male	No	0.58	0.38	Mann–Whitney	985	0.16	No
13	Male	No	5.64	5.96	Mann–Whitney	248,044	0.13	No
13	Male	No	11.01	14.08	Mann–Whitney	935,555	<10^−4^	Yes
13	Male	No	13.04	10.54	Mann–Whitney	778,029	1.9 × 10^−3^	Yes
9	Female	No	0.24	0.85	Mann–Whitney	682	<10^−4^	Yes
11	Female	No	4.34	4.44	Mann–Whitney	113,683	0.37	No
11	Female	No	2.80	2.51	Mann–Whitney	46,063	0.31	No
16	Female	No	2.08	1.63	Mann–Whitney	23,424	0.05	No
8	Male	Yes	0.46	1.42	Mann–Whitney	2,676	<10^−4^	Yes
11	Male	Yes	3.73	7.50	Mann–Whitney	95,475	<10^−4^	Yes
12	Male	Yes	0.28	0.46	Mann–Whitney	729	7.0 × 10^−3^	Yes
7	Female	Yes	0.15	1.79	Mann–Whitney	328	<10^−4^	Yes
16	Female	Yes	6.14	7.17	Mann–Whitney	282,120	0.15	No
16	Female	Yes	0.31	0.93	Mann–Whitney	842	2 × 10^−4^	Yes

**Table 4.** Amplitude of sEPSCs by neuron (RS neurons)

Age (PND)	Sex	Gabazine	Baseline median amplitude (pA)	Nicotine median amplitude (pA)	Statistical test	*U*	*p* value	*p* < 0.05
8	Male	No	16.71	16.61	Mann–Whitney	736,431	0.72	No
8	Male	No	11.15	13.32	Mann–Whitney	2,069	8.5 × 10^−3^	Yes
11	Male	No	18.27	17.58	Mann–Whitney	293,017	0.10	No
12	Male	No	22.72	18.19	Mann–Whitney	9,421	<10^−4^	Yes
12	Male	No	14.85	13.25	Mann–Whitney	2,785	0.33	No
13	Male	No	15.78	16.19	Mann–Whitney	252,739	0.33	No
13	Male	No	24.26	19.18	Mann–Whitney	857,147	<10^−4^	Yes
13	Male	No	15.44	20.79	Mann–Whitney	663,733	<10^−4^	Yes
9	Female	No	18.04	28.15	Mann–Whitney	1,284	0.07	No
11	Female	No	15.23	18.78	Mann–Whitney	104,126	1.5 × 10^−3^	Yes
11	Female	No	13.63	13.65	Mann–Whitney	47,813	0.81	No
16	Female	No	13.72	12.62	Mann–Whitney	24,020	0.13	No
8	Male	Yes	14.54	20.08	Mann–Whitney	4,018	<10^−4^	Yes
12	Male	Yes	22.92	20.96	Mann–Whitney	1,107	0.83	No
16	Female	Yes	18.85	17.57	Mann–Whitney	267,705	2.0 × 10^−3^	Yes
16	Female	Yes	24.15	24.38	Mann–Whitney	1,371	0.43	No

We also calculated a median frequency and amplitude at baseline and with nicotine for each neuron and analyzed group effects with paired *t* tests. We tested for sex differences in each experimental group where powered and grouped animals according to age if none were found. Where relevant, figures depict sex of the animal for each associated data point. Parametric tests were used when assumptions of normality were met; otherwise, nonparametric tests were used.

## Results

### Identifying FS neurons

**Figure 1.**
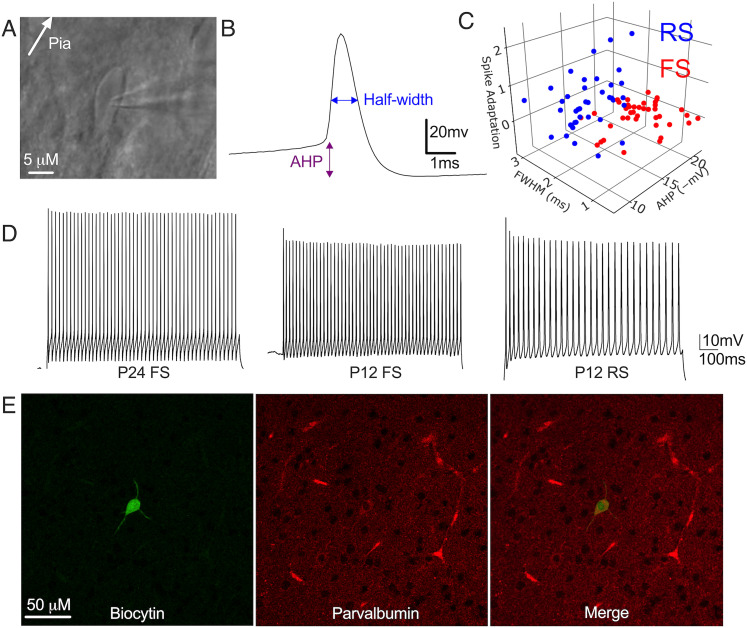
Identification of PV neurons in early development. ***A***, FS neuron imaged using differential interference contrast during whole-cell patch-clamp recording. ***B***, Recorded action potential from an FS neuron. Spike half-width and afterhyperpolarization (AHP) measurement locations indicated. ***C***, Distribution of kinetic parameters used to identify neurons as FS or RS [spike adaptation index, FWHM, full-width at half-maximum, or half-width (HW), and AHP]. Spike adaptation index is transformed by a scaling factor for simplicity of presentation. ***D***, Left, Trace of action potentials recorded from an FS neuron from an adult rat [AHP: −21.7 mV, HW: 0.6 ms, interevent interval (IEI) slope: 1.0 × 10^−5^]. Additional traces from neurons from P12 rats classified as FS (center; AHP: −17.9 mV, HW: 1.5 ms, IEI slope: 1.7 × 10^−4^) or RS (right; AHP: −14.9 mV, HW: 1.8 ms, IEI slope: 1.0 × 10^−3^). ***E***, Biocytin-filled recorded neuron positive for parvalbumin immunohistochemistry in a 300 μm section.

Figure 1*A* depicts a FS neuron as viewed through differential interference contrast (DIC) during live-slice electrophysiology with characteristic morphology including oblong shape and absence of a pronounced apical dendrite. FS neurons can be identified by the distinct properties of their action potentials, evoked by intracellular current injections (McCormick et al., 1985; Tateno et al., 2004; Subkhankulova et al., 2010). These include a brief spike duration, high firing frequency, a large afterhyperpolarization (AHP; Fig. 1*B*), and little or no spike frequency adaptation (Fig. 1*C*,*D*, left). We refer to neurons that meet these criteria as “FS neurons.” Whenever possible, we confirmed the identity of these neurons with post hoc parvalbumin (PV) immunohistochemistry and verification of absence of dendritic spines (Fig. 1*E*). We refer to neurons that express the calcium-binding protein PV as “PV neurons.” PV-expressing neurons account for the vast majority of FS cells in the neocortex (Kawaguchi and Kondo, 2002; Rudy et al., 2011).

The FS characteristics contrast with those of regular-spiking (RS) neurons that have longer spike durations, small or no AHP, and whose spike trains accommodate (Fig. 1*D*, right). RS cortical neurons may include excitatory neurons, inhibitory neurons, interneurons, or projection neurons that are not FS and do not express PV (Porter et al., 1999; Rudy et al., 2011). The RS phenotype encompasses substantial physiological variability, making subclassification based on firing patterns not feasible (Connors and Gutnick, 1990; Gottlieb and Keller, 1997).

It is more challenging to identify FS neurons in younger animals in which currents that drive the distinctive firing characteristics are still developing (Okaty et al., 2009; Subkhankulova et al., 2010) and in which significant PV expression begins only around P12 (Alcántara et al., 1993; De Lecea et al., 1995; Itami et al., 2007). To address this, we developed quantitative criteria to distinguish between FS and RS neurons. These criteria were obtained from recordings of 87 neurons from animals ranging in age from P7 to P26 (Fig. 1*C*) and were validated by IHC in a subset of cells (Fig. 1*E*). Neurons were identified as FS if they had AHP less than or equal to −10 mV, action potential width (at half maximum) ≤1.7 ms, and slope of interspike intervals (IEI slope, or spike adaptation index) ≤7.1 × 10^−4^. Neurons that did not meet these criteria were classified as RS neurons. Figure 1*D* depicts the differences in action potential kinetics obtained from P12 neurons classified as FS and RS, respectively. The depicted P12 FS neuron has an AHP of −17.9 mV, half-width of 1.5 ms, and IEI slope of 1.7 × 10^−4^, while the RS neuron has a longer half-width (1.8 ms) and more pronounced spike adaptation (IEI slope = 1.0 × 10^−3^).

### Nicotine increases excitatory synaptic inputs to FS neurons in S1 across development

nAChRs are expressed by inhibitory, GABAergic cortical neurons, and by the presynaptic, glutamatergic terminals that provide excitatory inputs to these neurons (Gil et al., 1997; Demars and Morishita, 2014; Askew et al., 2019; Gil and Metherate, 2019). Previous studies examined the effects of nicotine on FS neurons after the second postnatal week and into adulthood. Therefore, we focused on comparing the second postnatal week (P8–P14) and the third week and beyond (P15–26). The second postnatal week in rats coincides with biophysical and chemical maturation of FS neurons (Itami et al., 2007) and a number of other important developmental changes. We investigated whether nAChR activation would increase synaptic drive onto FS neurons in early postnatal development and compared this to responses in early adolescence.

**Figure 2.**
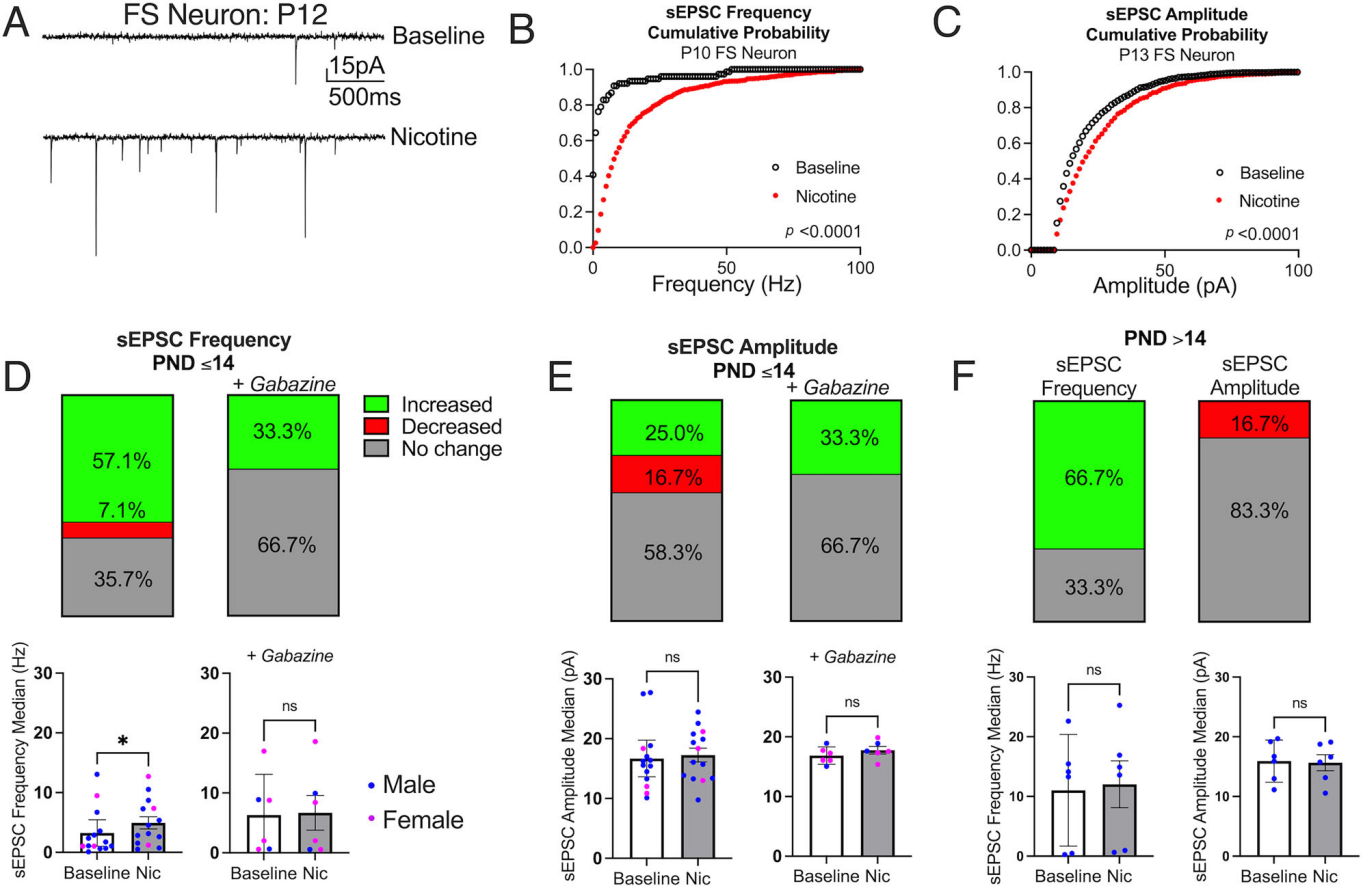
Nicotine enhances excitatory inputs to FS neurons. ***A***, Spontaneous excitatory postsynaptic currents (sEPSCs) recorded from a P12 FS neuron that responded to nicotine by increasing frequency and amplitude of sEPSCs. ***B***, Cumulative probability distribution of sEPSC frequency from a P10 FS neuron (Mann–Whitney test, *U* = 6,588, *p* < 0.0001). ***C***, Cumulative probability distribution of sEPSC amplitude from a P13 FS neuron (Mann–Whitney test, *U* = 669,189, *p* < 0.0001). Percentages of FS neurons from P10 to 14 animals that significantly increased frequency (***D***, *n* = 14 neurons without gabazine; *n* = 6 neurons with gabazine; see Table 1 for individual statistical tests) and amplitude (***E***, *n* = 12 neurons without gabazine; *n* = 6 neurons with gabazine; see Table 2 for individual statistical tests) of sEPSCs with nicotine. Individual medians of sEPSC frequency after nicotine application in the absence of (paired *t* test, *t*_(13)_ = 2.33, *p* = 0.04, Cohen's *d* = 0.62) and presence of (paired *t* test, *t*_(5)_ = 1.34, *p* = 0.31) gabazine are shown in ***D***, bottom panels. Individual medians of sEPSC amplitude after nicotine application in the absence of (paired *t* test, *t*_(13)_ = 0.49, *p* = 0.63) and presence of (paired *t* test, *t*_(5)_ = 1.50, *p* = 0.19) gabazine are in ***E***, bottom panels. ***F***. Percentages of FS neurons from P15–26 animals that significantly increased frequency or decreased amplitude of sEPSCs with nicotine (*n* = 6 neurons; see Tables 1, 2 for individual statistical tests). Individual medians of sEPSC frequency (bottom left, paired *t* test, *t*_(5)_ = 1.54, *p* = 0.18) and sEPSC amplitude (bottom right, paired *t* test, *t*_(5)_ = 2.16, *p* = 0.08). Mean ± 95% CI (***D***, ***E***, ***F***, bottom panels).

We recorded sEPSCs in S1 layer 4 FS neurons from postnatal rats (*N* = 19 rats P10–26, *n* = 1–2 neurons per rat). We assessed the frequency and amplitude of events from 3 min recordings at baseline in ACSF and after a 3 min wash-in of 10 µM nicotine. Frequency and amplitude of events were increased in some neurons by application of nicotine. Figure 2*A* depicts traces of sEPSCs before and after nicotine application recorded from a FS neuron that significantly increased frequency and amplitude during the second postnatal week. A representative cumulative probability distribution curve for a neuron that responded to nicotine with an increased frequency of sEPSCs is shown in Figure 2*B* and for a neuron that responded to nicotine with increased amplitude of sEPSCs is shown in Figure 2*C*. Nicotine produced significant rightward shifts in the cumulative probability distribution of frequency and amplitude, respectively, of each neuron depicted. Most (57%) neurons in the younger age group showed an increase in frequency (Fig. 2*D*, top left), and 25% exhibited an increase in sEPSC amplitude (Fig. 2*E*, top left). In 7% of recorded FS neurons in younger animals, frequency and amplitude increased (see Tables 1, 2 for individual statistical tests). Nicotine significantly increased the median frequency of sEPSCs in group analysis (Fig. 2*D*, bottom left), but not amplitude (Fig. 2*E*, bottom left).

In a subset of recordings from FS neurons in P10–14 rats, we included the GABA-A receptor antagonist, gabazine (1 µM), in the ACSF to suppress effects of inhibitory interneurons on the recorded cells. In this condition, similar ratios of FS neurons responded to nicotine with an increased frequency (33.3%; Fig. 2*D*, top right) and amplitude (33.3%; Fig. 2*E*, top right). However, the group-level enhancement of frequency was abolished when gabazine was included in the recording solution (Fig. 2*D*, bottom right). Nicotine may partially be acting on inhibitory neurons to drive increases in sEPSC frequency in FS neurons at young ages. As some of these recordings occurred before GABA currents switch from excitatory to inhibitory, a developmental milestone that occurs by the end of the second postnatal week (Peerboom and Wierenga, 2021), the increased frequency from nicotine application at early ages may be directly driven by excitatory GABA signaling. We have also considered an indirect mechanism involving disinhibition via other interneurons, as has been proposed in other studies (Askew, et al., 2019).

We also investigated effects of nicotine on frequency and amplitude of sEPSCs in RS neurons in young animals. Nicotine significantly increased sEPSC frequency in 62.5% of the recorded RS neurons (*n* = 16 neurons; data not shown: see Tables 3, 4 for individual statistical tests) and increased sEPSC amplitude in approximately one-quarter of them (27.3%; *n* = 11 neurons; data not shown: see Tables 3, 4 for individual statistical tests). When indirect effects on inhibitory neurons were suppressed with gabazine, nicotine increased sEPSC frequency in all RS neurons (*n* = 4 neurons; data not shown: see Table 3 for individual statistical tests).

In older animals (P15–26), there were no group effects of nicotine on either the frequency (Fig. 2*F*, bottom left) or amplitude (Fig. 2*F*, bottom right) of sEPSCs. While a similar proportion of neurons in this older age group still showed increased sEPSC frequency (Fig. 2*F*, top left), none of the neurons responded with an increase in amplitude at these ages (Fig. 2*F*, top right). These results collectively indicate that while nicotine increases the frequency of excitatory synaptic events in a majority of FS neurons across development (from the second postnatal week into adolescence), the group-level impact on frequency is primarily evident in younger animals, and the amplitude-enhancing effects are specifically limited to this early developmental period in a smaller subset of FS neurons.

### Nicotine does not produce whole-cell currents in FS neurons in S1

We also explored direct postsynaptic effects of nicotine on FS neurons in S1, beginning in early development. While previous research in adult rodents has generally reported that fast-spiking (FS) neurons do not demonstrate direct postsynaptic nicotinic currents in various cortical regions in adults, including prefrontal cortex (Couey et al., 2007; Gulledge et al., 2007), motor cortex (Porter et al., 1999), visual cortex and somatosensory cortex (Gulledge et al., 2007), our study specifically investigates these effects in earlier developmental periods in S1. In contrast to FS neurons, regular-spiking (RS) neurons across multiple cortical areas typically depolarize in response to nicotine, whether applied via bath (Askew et al., 2019) or directly to the soma (Couey et al., 2007; Gulledge et al., 2007). Consequently, some have suggested that FS neurons may not possess functional nAChRs in rodents (Couey et al., 2007; Askew et al., 2019). There is some evidence from other species demonstrating expression of nAChR subunits in PV neurons (Krenz et al., 2001; Disney et al., 2007), though to our knowledge this has not been explored in rats.

**Figure 3.**
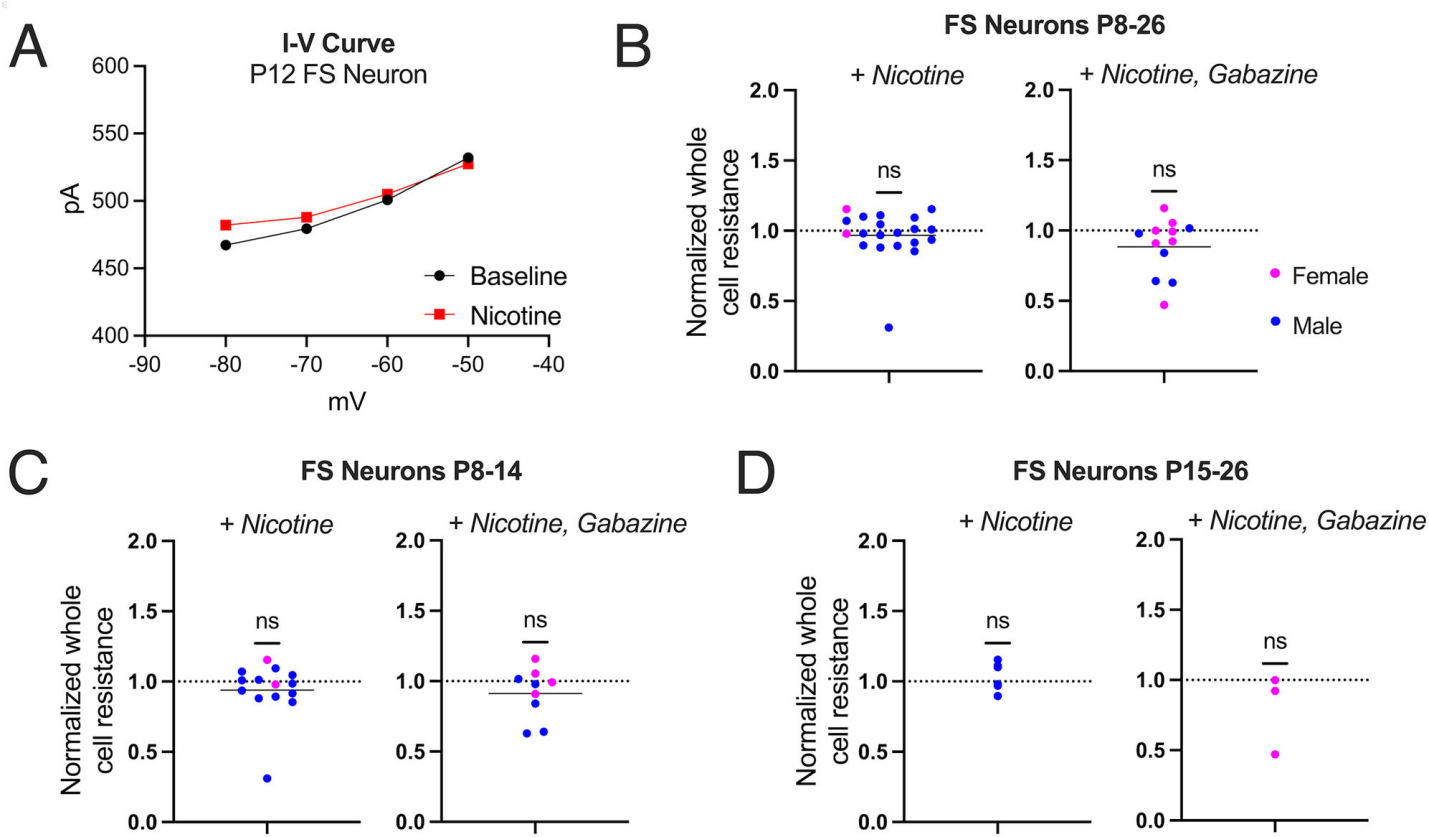
Somatodendritic responses of FS neurons in S1. ***A***, *I*–*V* curve of a P12 FS neuron before and after nicotine application. ***B***, Normalized whole-cell resistance of all FS neurons after nicotine application (left, *n* = 20, paired *t* test, *t*_(19)_ = 0.77, *p* = 0.45) and in the presence of gabazine (right, *n* = 12, paired *t* test, *t*_(11)_ = 1.81, *p* = 0.10). ***C***, Normalized whole-cell resistance of FS neurons from P8–14 rats (left, *n* = 14, paired *t* test, *t*_(13)_ = 0.77, *p* = 0.45) and with gabazine (right, *n* = 9, paired *t* test, *t*_(8)_ = 1.56, *p* = 0.16). ***D***, Normalized whole-cell resistance of FS neurons from P15–26 rats (left, *n* = 6, paired *t* test, *t*_(5)_ = 0.05, *p* = 0.96) and with gabazine (right, *n* = 3, paired *t* test, *t*_(2)_ = 1.18, *p* = 0.36).

We hypothesized that nicotine may have postsynaptic effects on FS neurons at very young ages and again focused on specific differences in effects between the second postnatal week and after P15. We calculated normalized neuronal resistance changes, an indirect measure of whole-cell currents, after applying nicotine (10 µM) for 3 min. The representative current–voltage (*I*–*V*) relationship shown in Figure 3*A* illustrates that nicotine application did not affect currents across the tested voltage range in an example FS neuron. Nicotine application did not decrease whole-cell resistance in FS neurons at any age range examined, from P8–26 (Fig. 3*B*, left), P8–14 (Fig. 3*C*, left), or P15–26 (Fig. 3*F*, left). These experiments indicate that FS neurons in S1 lack direct postsynaptic nicotinic responses across all developmental stages investigated, indicating that nicotinic modulation of these critical inhibitory neurons likely occurs through other mechanisms.

### Absence of nicotinic responses in FS neurons is not attributable to receptor desensitization

**Figure 4.**
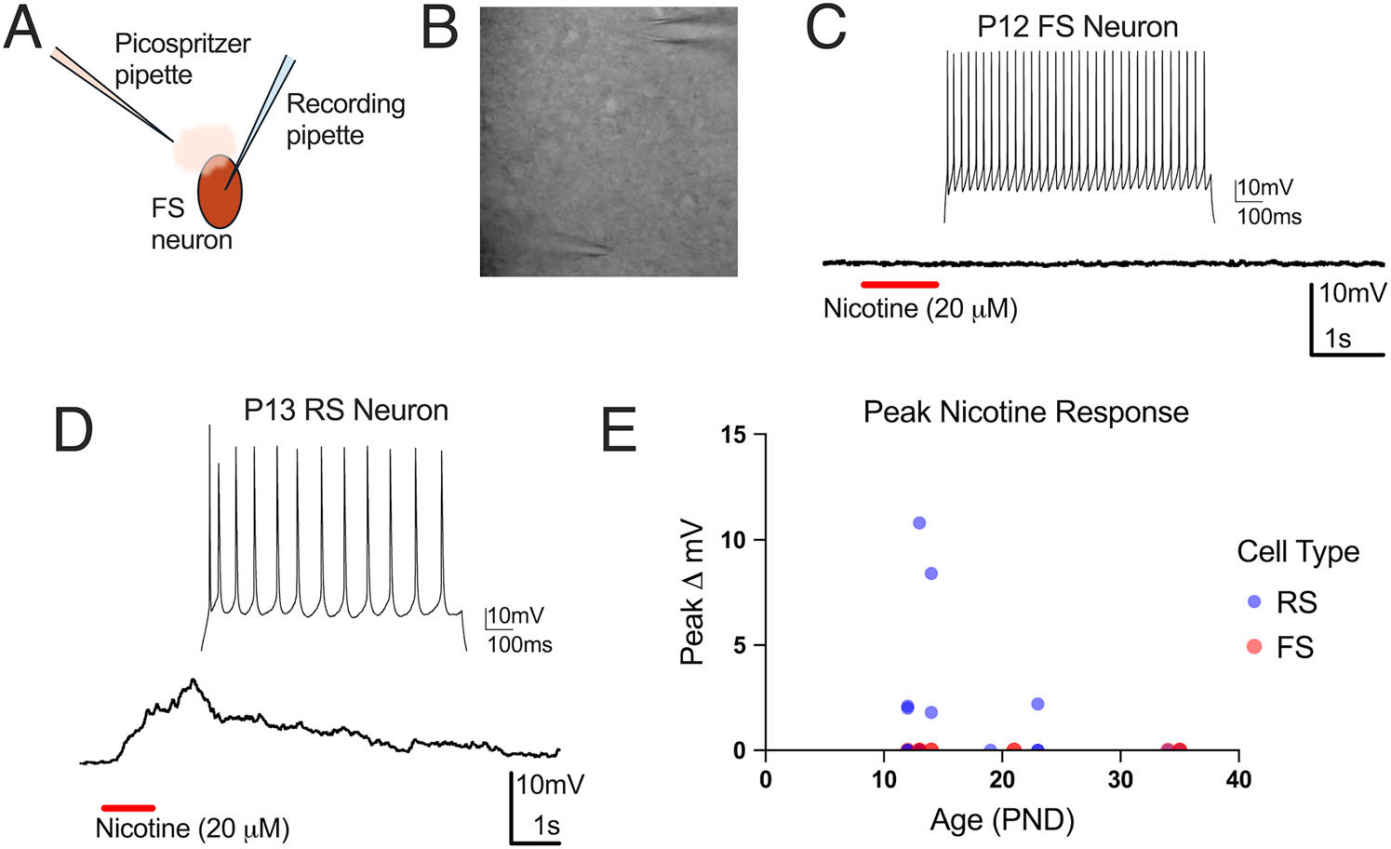
FS and RS responses to direct nicotine application. ***A***, Schematic for nicotine puff recordings. ***B***, Image of puff recording with recording pipette (top) and puff pipette (bottom). ***C***, Representative recording from a FS neuron with direct nicotine application. ***D***, Representative recording from a RS neuron that depolarized in response to direct nicotine application (average of 5 responses). ***E***, Summary of voltage changes from RS and FS neurons with direct nicotine application at various ages (*N* = 10 animals, *n* = 10 FS neurons; 18 RS neurons).

The sustained effects of nicotine bath application (10 µM, 3–6 min application) on inputs to FS neurons (Fig. 2) may represent a nondesensitizing nicotinic mechanism (Askew et al,., 2019). Our finding that bath-applied nicotine did not produce postsynaptic responses in FS neurons (Fig. 3) suggests that rapid receptor desensitization may preclude observation of postsynaptic nicotinic effects on these cells. To test this, we focally applied puffs of nicotine (20–30 µM) using a Picospritzer, while recording in current clamp in the presence of synaptic blockers (Fig. 4*A*,*B*). Nicotine did not depolarize any FS neuron at any age (Fig. 4*C*,*E*). In contrast, nicotine depolarized one-third of RS neurons by an average of 4.6 mV (Fig. 4*D*,*E*), in both younger and older pups. This result is consistent with previous findings that nicotine induces small depolarizations in some RS neurons in sensory cortical areas (Askew et al., 2019), though previous literature has not demonstrated this effect in the presence of synaptic blockers. The lack of response in two-thirds of RS neurons to nicotine likely reflects the heterogeneous and cell type-specific distribution of functional nAChRs within cortical circuits (Couey et al., 2007; Gulledge et al., 2007; Arroyo et al., 2014; Askew et al., 2019). Many RS neurons, particularly pyramidal cells, have sparse nAChRs and are often weakly or indirectly depolarized by nicotine (Askew et al., 2019). As this brief nicotine application method reduces the likelihood of receptor desensitization, this suggests that FS neurons lack postsynaptic responses to nicotine. These results demonstrate that FS neurons do not have postsynaptic responses to nicotine applied via bath or directly to the soma, as early as these neurons can be reliably identified through early adolescence.

### FS neurons express two common nicotinic receptor subunits

**Figure 5.**
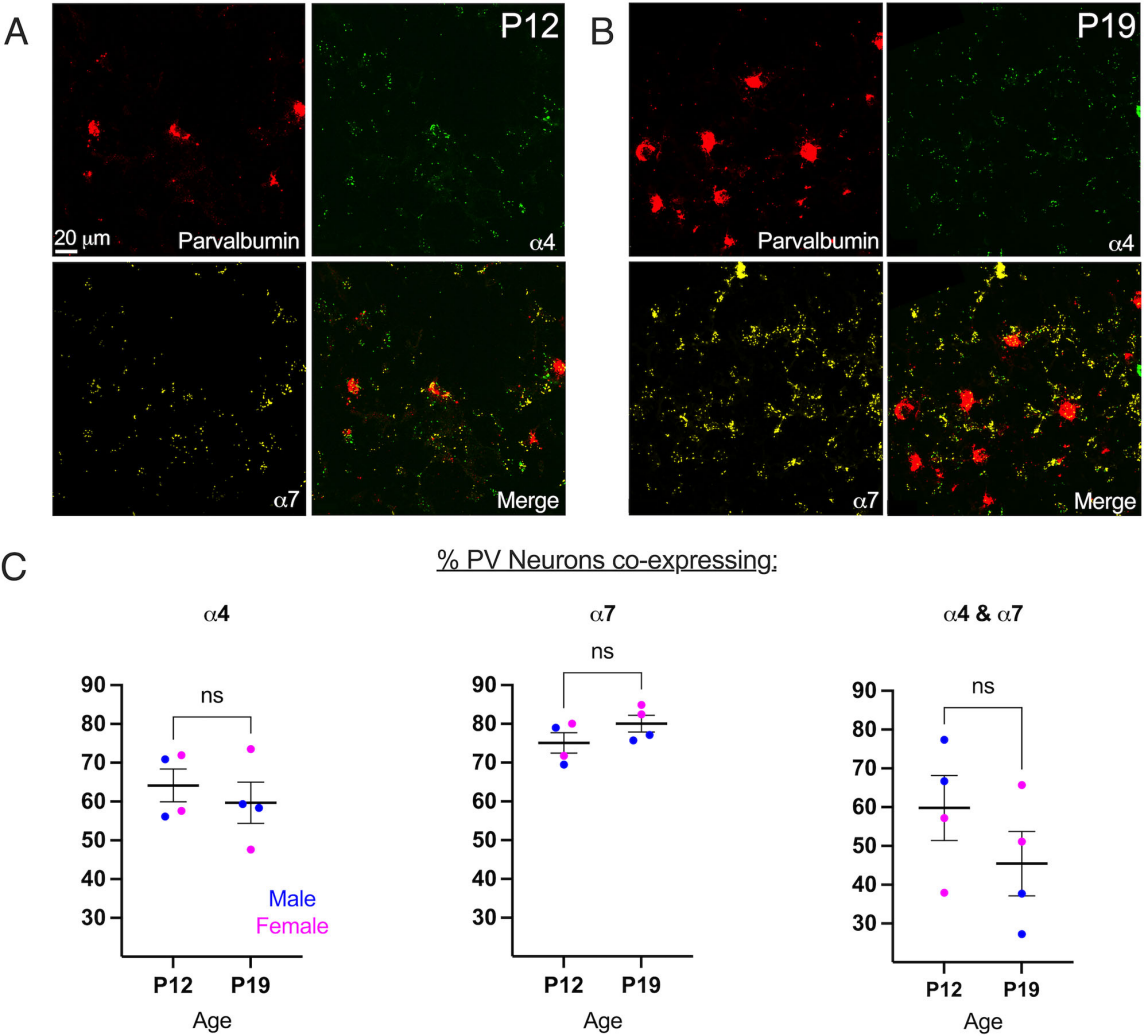
PV neurons express mRNAs that encode multiple nAChR subunits in S1: α4 and α7. RNAscope images from S1 in a P12 (***A***) and P19 (***B***) rat showing coexpression of α4 and α7 mRNA in neurons also expressing PV. ***C***, Coexpression levels of nAChR mRNA in PV neurons from P12 and P19 rats (left, α4, *N* = 2 rats/age, *n*_P12_ = 1,910 PV neurons, *n*_P19_ = 2,946 PV neurons, unpaired *t* test, *t*_(6)_ = 0.65, *p* = 0.54; center, α7: *N* = 2 rats/age, *n*_P12_ = 1,910 PV neurons, *n*_P19_ = 2,946 PV neurons, unpaired *t* test, *t*_(6)_ = 1.46, *p* = 0.20; right, α4* and α7: *N* = 2 rats/age, *n*_P12_ = 1,910 PV neurons, *n*_P19_ = 2,946 PV neurons, unpaired *t* test, *t*_(6)_ = 1.22, *p* = 0.27). Mean ± 95% CI (***C***).

The α4β2 heteromeric and α7 homomeric nAChRs are the most commonly expressed nicotinic receptors in mammalian cortex (Millar and Gotti, 2009; Nair and Liu, 2019). Figure 5, *A* and *B*, depicts images of sections through the barrel cortex of P12 rats, demonstrating localization of mRNA for PV and for CHRNA4 and CHRNA7, the genes coding for the α4 and α7 nicotinic subunits, respectively. At P12, 64.2% of PV neurons expressed CHRNA4, and 75.1% expressed CHRNA7 (Fig. 5*C*). More than half of PV neurons (59.8%) at P12 coexpressed mRNA that encode α4 and α7 nAChR subunits (Fig. 5*C*, right). At P19, 59.7% of PV neurons expressed α4 nAChR-encoding mRNA, 80.0% expressed α7-nAChR-encoding mRNA, and 45.4% coexpressed both mRNAs (Fig. 5*C*). These expression levels did not significantly differ between P12 and P19. These data indicate that PV neurons express mRNAs that encode α4 and α7 nAChR subunits from early in development, near the time point when these neurons develop characteristic fast-spiking properties.

### Lynx1 expression increases early in development in S1

The expression of mRNAs that encode nAChR subunits by PV neurons, including in younger animals, contrasts with the lack of responses of these cells to nicotine. One mechanism that may prevent activation of nAChRs is the expression of lynx1 protein. Lynx1 is a nAChR modulator coexpressed with nAChRs in the brain (Ibañez-Tallon et al., 2002). Lynx1 acts as a molecular brake on nicotinic signaling by orthosterically binding nAChRs, thereby limiting the elevated levels of cortical plasticity observed during developmental critical periods to their more stable, characteristic adult states (Morishita et al., 2010; Takesian et al., 2018; Miwa, 2021). Studies of lynx1 control of plasticity have focused on auditory (Takesian et al., 2018) or visual cortex (Morishita et al., 2010; Bukhari et al., 2015; Sadahiro et al., 2016; Sajo et al., 2016). The timeline of lynx1 expression and its role in the somatosensory cortex is not known.

**Figure 6.**
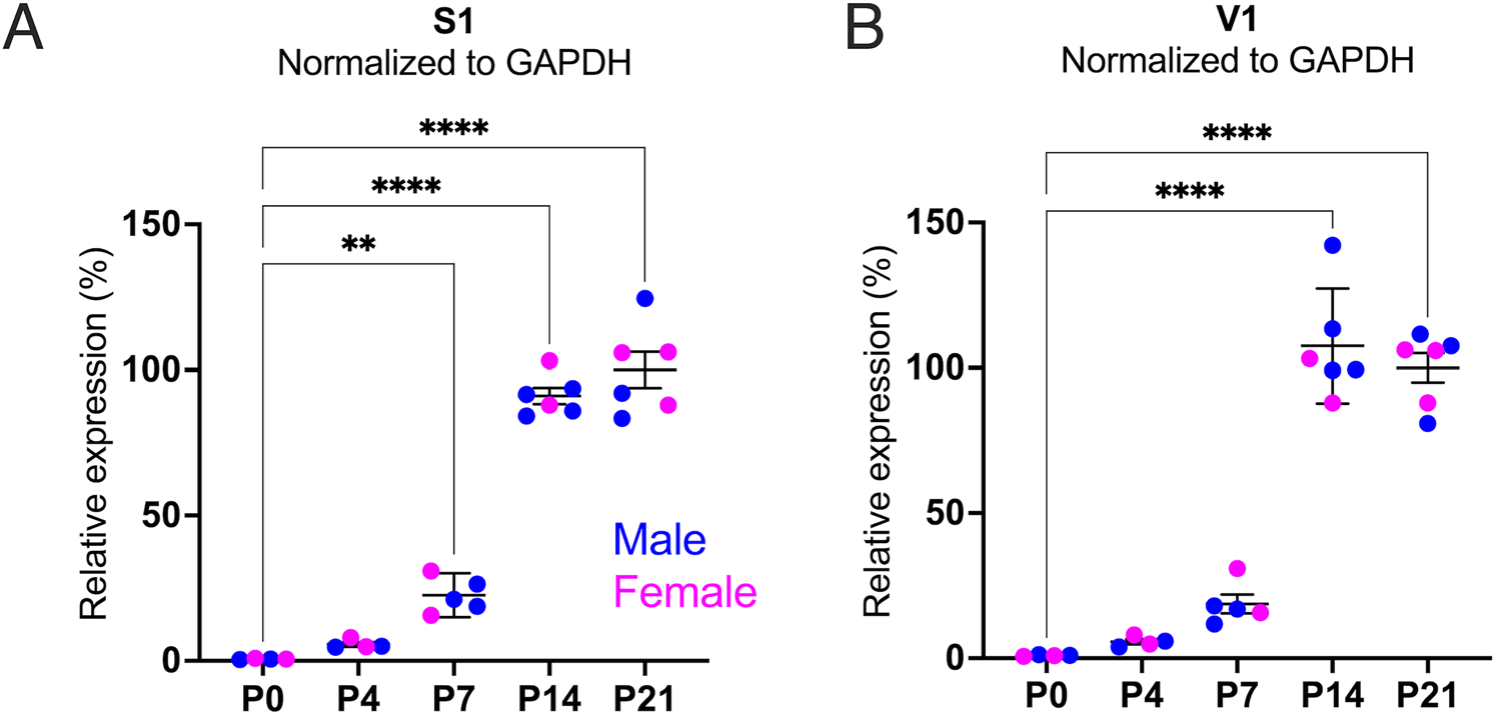
*Lynx1* expression increases in S1 and V1 in the second postnatal week. Relative mRNA expression levels of *lynx1* in S1 (***A***, *n* = 4–8 animals at each age, *F*_(4,20)_ = 150.4, *p* < 0.0001, one-way ANOVA, Dunnett's multiple-comparison tests) and V1 (***B***, *n* = 4–8 animals at each age, *F*_(4,20)_ = 102.7, *p* < 0.0001, one-way ANOVA, Dunnett's multiple-comparison tests). Mean ± 95% CI (***A***, ***B***).

We examined whether lynx1 is expressed in the developing S1 cortex and compared this expression with that in the visual cortex. We used RT-qPCR to quantify *lynx1* mRNA levels in rat S1 and primary visual cortex (V1) across developmental ages. *Lynx1* mRNA was not detectable in neonates (P0), underwent a gradual increase in the first postnatal week (P7), and greatly increased in the second postnatal week (Fig. 6*A*). In V1, *lynx1* mRNA expression profile was similar, as the greatest increase in mRNA expression occurred during the second postnatal week (Fig. 6*B*).

**Figure 7.**
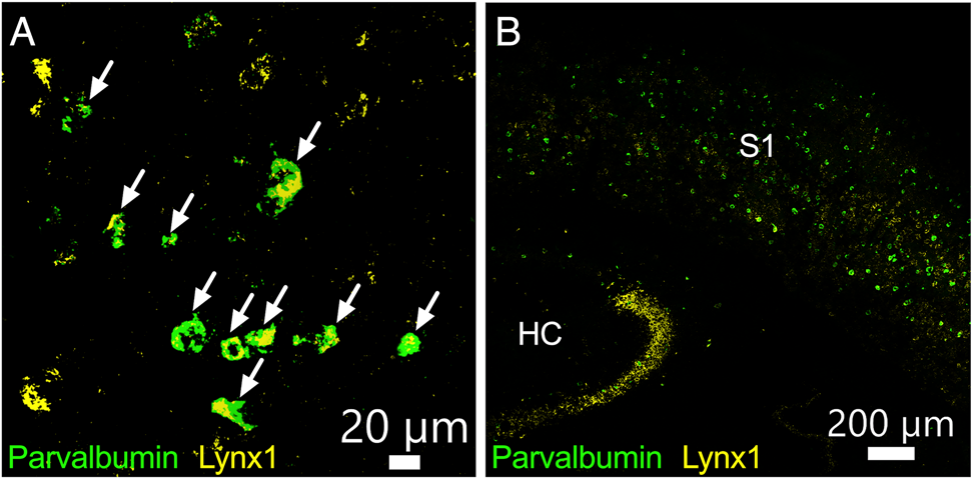
Lynx1-encoding mRNA is expressed in PV neurons in mice early in development through adulthood. RNAscope images from S1 in a P12 (***A***) and P100 (***B***) mouse showing coexpression of *lynx1* mRNA in neurons also expressing parvalbumin. Characteristic *lynx1* enrichment in CA2/3 region of adult hippocampus (Miwa et al., 1999) is shown in (***B***) HC, hippocampus; S1, primary somatosensory cortex. See Results for expression levels.

To examine the expression of *lynx1* mRNA specifically in PV neurons we took advantage of in situ hybridization (RNAscope). We studied mouse S1 during the second postnatal week (P12; Fig. 7*A*) and during adulthood (P100; Fig. 7*B*). We focused on the posteromedial barrel subfield of S1 and analyzed all cortical layers (Woolsey and Van der Loos, 1970). At P12, 96.4% of PV neurons expressed *lynx1* (data not shown, *N* = 2 animals, *n* = 844 neurons), and at P100, 83.5% expressed *lynx1* (data not shown, *N* = 2 animals, *n* = 1,684 neurons), confirming that PV neurons express mRNA for a protein that can act as a brake on nicotinic receptor function from nearly as early as they can be reliably identified and recorded using electrophysiology. Coexpression of *lynx1* mRNA remains high in PV neurons well into adulthood. This high level of coexpression is consistent with previous findings in adult mice (Morishita et al., 2010; Demars and Morishita, 2014).

## Discussion

We established that nicotine, acting presynaptically, enhances excitatory inputs to FS neurons as early as the second postnatal week in rats, a period corresponding to late human gestation (Clancy et al., 2007). This is consistent with previous work demonstrating that nicotine or ACh, acting at nAChRs, increase frequency of excitatory inputs to adult cortical FS neurons (Couey et al., 2007; Kassam et al., 2008). The source of these inputs has not been identified, but intrinsic axon collaterals from corticothalamic neurons are a possible component (West et al., 2006; Kassam et al., 2008; Heath et al., 2010; Kim et al., 2014). In the present study, we successfully identified FS neurons at younger ages and found that nicotine increases the frequency of spontaneous excitatory postsynaptic currents (sEPSCs) in 57.1% of FS neurons from as early as P8.

The sensitivity to nicotine of synaptic inputs to FS neurons presents a unique vulnerability to exogenous nicotine exposure, such as from maternal smoking or vaping. These effects may be driven by a less well-characterized nicotinic receptor, such as the α4β2α5 receptor expressed in layer 6 pyramidal neurons that exhibits nicotinic responses that are highly resistant to desensitization, similar to the persistent effects on sEPSC frequency in our recordings (Heath et al., 2010; Bailey et al., 2014; Venkatesan et al., 2023). This receptor is implicated in many of the long-term changes resulting from nicotine exposure during prenatal or early postnatal development (Heath et al., 2010), and the CHRNA5 gene coding for the α5 nAChR subunit is linked to nicotine addiction, schizophrenia, and effects of developmental nicotine exposure (Hong et al., 2011; Bailey et al., 2014).

In contrast to the effects on presynaptic inputs, we found no evidence for nicotine having direct effects on the somatodendritic compartment of FS neurons at any time during development. The results in older animals are consistent with previous literature demonstrating that nicotinic agonists do not depolarize FS neurons in adults (Porter et al., 1999; Couey et al., 2007; Gulledge et al., 2007). We now show that FS neurons from even younger animals—during the second postnatal week—do not respond to nicotine.

To determine if the lack of responses is related to the expression—or lack thereof—of nicotinic receptors, we established a developmental timeline of expression of mRNAs encoding the α4 and α7 nAChR subunits, the most commonly expressed in mammalian cortex (Millar and Gotti, 2009; Nair and Liu, 2019), in S1 PV neurons. Both receptors are developmentally regulated (Naeff et al., 1992). We established that, early in development, nearly two-thirds of PV neurons in rat S1 express the gene coding for α4 nAChR subunits and three-fourths of them express the gene coding for α7 nAChR subunits. More than half express mRNAs encoding both receptor subunits. This reveals a heterogeneity in expression of nAChR subunits that may correspond to different PV neuron subtypes, such as basket and chandelier cells. Very few PV neurons expressed neither mRNA-encoding receptor subunit. Despite the known early upregulation of these mRNAs in cortical neurons (Naeff et al., 1992), the expression profile in PV neurons changed little between P12 and P19 in our analysis.

The expression of these nAChR subunits in PV neurons, their early emergence in development, and relatively stable expression contrasts with the lack of responses of these neurons to nicotinic stimulation. We considered possible mechanisms for this contradiction. Whether nAChRs in PV neurons are expressed in somatodendritic compartments or in axon terminals has not been established. Nicotine enhances frequency and amplitude of spontaneous inhibitory postsynaptic currents (sIPSCs) in cortical pyramidal neurons, possibly through effects on 5HT3AR+ inhibitory neurons (Couey et al., 2007; Takesian et al., 2018; Askew et al., 2019). Further work is needed to uncover whether α4β2, α7, α4β2α5, or other nAChRs play a role in transmitter release at the terminals of FS neurons. To dissect how nicotinic signaling influences cortical inhibitory circuits and to complement our sEPSC findings, future studies should investigate the direct and indirect effects of nicotine on sIPSCs in cortical neurons during early development, particularly considering our observation that gabazine abolished the group-level sEPSC frequency enhancement in FS neurons.

To isolate direct cellular responses to nicotine, we suppressed other receptors with a cocktail of ionotropic glutamatergic (AP5 and CNQX) and GABAergic (gabazine) receptor antagonists. While effective in blocking fast ionotropic synaptic transmission, we acknowledge that this approach does not eliminate all potential indirect effects (Porter et al., 1999; Askew et al., 2019). We did not use tetrodotoxin (TTX), a voltage-dependent sodium channel blocker, in these experiments because that would have suppressed neuronal firing, which is necessary to identify FS neurons by their distinct firing patterns.

Lynx1, a prototoxin that serves as a brake on nicotinic signaling in other cortical areas (Morishita et al., 2010), is expressed in PV neurons, but its role in these neurons is unclear. Lynx1 is part of the large Ly6/uPAR/neurotoxin superfamily whose members are often expressed alongside nAChRs as accessory proteins (Miwa, 2021). Lynx1 is transported to the cellular membrane, binds to nAChRs, and reduces their signaling in a similar manner to toxins in elapid snake venom (Miwa et al., 1999). We revealed a large developmental increase in expression of the mRNA that encodes lynx1 in S1 and confirmed that it is expressed in PV neurons in the second postnatal week.

Changes in the expression of lynx1 across development have been studied only in visual and auditory cortex and only at later developmental stages (≥P18). Here, we provide the first analyses of lynx1 expression starting at birth, in both V1 and S1. We find that, in S1, *lynx1* mRNA expression is not detectable in neonates but increases sharply around P7. In V1, *lynx1* mRNA expression profile was similar, as the greatest increase in mRNA expression occurred during the second postnatal week.

Mechanistic studies of the functions of lynx1 are complicated by its molecular structure. The native form of lynx1 is a glycophosphatidylinositol-anchored membrane protein (Anderson et al., 2020), which poses considerable difficulties for in vitro studies and direct experimental manipulation. It is difficult to synthesize recombinant lynx1 protein without introducing modifications to its structure that make it water-soluble, thereby changing its binding and functional properties (Lyukmanova et al., 2011). There are also no reliable antibodies for immunohistochemistry. Similar issues exist with antibodies for nAChR subunits (Moser et al., 2007). Consequently, to accurately assess developmental timelines of lynx1 and nAChR subunit expression, this work primarily relied on transcriptional level analyses. While such methods provide valuable insights into gene expression, a direct correlation between mRNA levels and protein concentrations is not always guaranteed.

The RNAscope assays for *lynx1* mRNA expression in PV neurons were performed using mouse tissue, whereas other data were obtained from rats because there were no validated RNAscope probes for rat *lynx1* at the time of the study. We recognize that extrapolating data between mice and rats presents complexities. While both are widely used models in neurodevelopmental research, there are recognized differences in their developmental timelines, gestational periods, and rates of maturation. Although general patterns of nAChR subunit distribution are largely conserved across vertebrates, subtle species-specific differences may exist (Millar and Gotti, 2009). Therefore, while our mouse RNAscope data provide valuable insights into *lynx1* expression in PV neurons, this species difference should be considered a caveat when interpreting the developmental changes or direct quantitative comparisons of *lynx1* mRNA expression relative to the other rat-derived data.

The sharp increase in *lynx1* expression suggests that, at earlier ages, when S1 is undergoing rapid development, FS neurons may be regulated by nicotinic currents when *lynx1* expression is low or absent. Because FS neurons in rats cannot be reliably identified this early (see above), we could not test this hypothesis directly. Developmental changes in lynx1 regulation of nicotinic responses may be involved in shaping plasticity and critical periods in S1 development (Erzurumlu and Gaspar, 2012). In V1, *lynx1* mRNA expression increases at the end of the critical period for monocular deprivation (Morishita et al., 2010). In mice lacking the *lynx1* gene, this window of plasticity remains open well into adulthood (Morishita et al., 2010). Converging evidence implicates lynx1 in the closure of critical periods across sensory cortex, with a different developmental profile in each (Anderson et al., 2020). Lynx1 may be an important nicotinic plasticity brake in multiple cortical areas, which presents treatment possibilities for perinatal nicotine exposure, as well as other cognitive and developmental disorders that involve impaired cortical plasticity. Its role and mechanism in FS neurons during the first 2 weeks of development remains to be explored. Nicotinic dysfunction in FS neurons is associated with numerous neuropsychological and neurological conditions, but nicotinic mechanisms driving development of FS neurons are understudied.

## Synthesis

Reviewing Editor: Lindsay De Biase, University of California Los Angeles Decisions are customarily a result of the Reviewing Editor and the peer reviewers coming together and discussing their recommendations until a consensus is reached. When revisions are invited, a fact-based synthesis statement explaining their decision and outlining what is needed to prepare a revision will be listed below. The following reviewer(s) agreed to reveal their identity: NONE.

Overall the reviewers found the manuscript of value, judged the experimental design and statistical analyses to be appropriate and straightforward. They outlined a number of limitations and clarifications that should be addressed in a revision:

1. An explanation should be provided for the following:

a. why the Lynx1 RNAscope analysis was done in mice whereas the ion channel expression as well as RT-qPCR Lynx1 analysis was done in rat. If this design was limited by availability of RNAscope probes, this should be stated and discussed as a caveat.

b. On page 19, the authors mention that nicotine depolarizes 1/3 of RS. neurons. Can the author speculate why the other 2/3 do not respond?

c. In figure 7, it looks like nearly everything is expressing Lynx1. Could the authors comments to what extent there are Lynx1negative cells in S1?

2. All expression analyses are restricted to the mRNA level, which does not need to proportionally translate to protein expression. If it is not possible to corroborate this at the protein level, some discussion of this should be included in the manuscript.

3. To study direct effects of nicotine on recorded cells a cocktail of ionotropic glutamatergic and GABAergic receptor antagonists was applied. This could have possible effect of metabotropic receptor activation or the activation of receptors by the release of non-glutamate/GABA neurotransmitters (e.g. neuromodulators). The addition of tetrodotoxin would have been useful. A discussion of this limitation would be sufficient.

4. Although the focus here is on sEPCS there are discrepancies whether the authors recorded these in the presence of gabazine or not. The authors speculate this might result from GABAergic events that can be depolarizing at this age. The authors should investigate whether GABAergic events are impacted by nicotine to strengthen the impact of the manuscript and also help with the interpretation of their existing sEPSC data. Also in their discussion on page 28, the authors refer to evidence that the frequency and amplitude of spontaneous IPSCs can be modulated by nicotine.

5. Some additional data would strengthen the manuscript

a. The authors conclude that nicotine does not affect the resting membrane potential or rheobase of FS interneurons, but this is only based on any 3 neurons. This is too low of a 'n' to make any clear conclusions.

b. Similarly, the authors suggest that nicotine application does not increase the spike frequency of FSI neurons based on the same neurons. Again, this is a low 'n' - furthermore the spiking plots shown for middle neuron in Fig. 3E looks a bit strange. Also, would it not be good to make an average out of these three plots

6. Additional clarification is needed for the following

a. It is unclear how whole-cell resistances, and many other electrophysiological parameters are calculated. This can be clarified further in Methods. Instead of normalized cell resistance as in Figure 3 it would be good to have the actual resistance measures.

b. It would be good to show the impact of nicotine on the sEPSCs in RS neurons. Also, in Figure 3 the title suggest that data is depicted of both FS and RS but only FS is given? Bit unclear when experiments were done on RS or not and when these are reported in manuscript.

Minor points

1. Double-check the description of dilutions of antibodies - 1:500 is unusual for a secondary antibody.

2. Whenever a percentage of frequency is stated, in addition to number of sections and animals, a total number of cells investigated should be mentioned.

3. With "DIC", do the authors really mean Diffusion Interference Contrast (then they would need to explain what this is) or Differential Interference Contrast?

4. Wouldn't they want to abbreviate "full width at half maximum" with FWHM?

5. Are the RS neurons in their dataset excitatory neurons or interneurons?

6. Figure 2: What is the definition of no change in spontaneous EPSC frequency?

7. In figure 2, it seems that in the PND>14 there are two groups which either receive high frequency inputs or those that do not receive any inputs. Could the authors speculate why this might be?

8. The author should include an elaboration to what extent their FS interneurons as defined electrophysiologically correspond to PV, CCK or other FS interneurons, as the authors use these terms interchangeably, and it might be a heterogeneous group.

9. For figure 7, it would be useful to understand how the authors delineate S1 and how many neurons were counted in these sections.

Additional reviewer comments that the authors may want to address in a response. While this may be beyond the scope of the Ms, it would increase its impact, so this was left to the authors' discretion:

The authors tentatively suggest that low Lynx1 expression during early development might make it possible for nicotine to impact FS interneurons. This is an interesting speculation. The manuscript would greatly increase its impact if the authors could show that nicotine does affect FS neurons more greatly at younger ages, but they would need a way to identify these. Can this not be done even if only as a proof-of principle in a suitable transgenic rat or mouse that reveals the FS types using a fluorescent marker?
